# Use of prescription drugs and risk of postoperative red blood cell transfusion in breast cancer patients: a Danish population-based cohort study

**DOI:** 10.1186/s13058-017-0926-2

**Published:** 2017-12-22

**Authors:** Anne Marie L. Thomsen, Alma B. Pedersen, Nickolaj R. Kristensen, Bjarne Kuno Møller, Christian Erikstrup, Peer M. Christiansen, Mette Nørgaard, Deirdre Cronin-Fenton

**Affiliations:** 1 Department of Clinical Epidemiology, Aarhus University Hospital, Olof Palmes Allé 43-45, 8200 Aarhus N, Denmark; 2 Department of Clinical Immunology, Aarhus University Hospital, Palle Juul-Jensens Boulevard 99, 8200 Aarhus N, Denmark; 3 Breast Surgery Unit, Aarhus University Hospital/Randers Regional Hospital, Aarhus C, Denmark

**Keywords:** Breast cancer, Red blood cell transfusion, Aspirin, NSAIDs, SSRIs, Statins

## Abstract

**Background:**

Several frequently used prescription drugs may affect bleeding risk. We investigated use of aspirin, nonsteroidal anti-inflammatory drugs (NSAIDs), selective serotonin reuptake inhibitors (SSRIs), and statins and risk of postoperative red blood cell transfusion in breast cancer patients.

**Methods:**

Using Danish population-based registries, we identified a cohort of women who underwent surgery for primary breast cancer (*n* = 22,238) during 2005–2012 and ascertained their use of aspirin, NSAIDs, SSRIs, and statins. For each drug, patients were categorized as users if they filled ≥1 prescription in the 60 days prior to surgery. We calculated the 14-day risk of red blood cell transfusion and relative risks (RRs) with 95% confidence intervals (CIs), comparing users with nonusers for each drug and adjusting for age, cancer stage, and Charlson Comorbidity Index score.

**Results:**

In our cohort, 1385 (6.2%) women were aspirin users, 1794 (8.0%) were NSAID users, 1110 (4.9%) were SSRI users, and 2053 (9.1%) were statin users. The overall risk of red blood cell transfusion was 1.3%. The 14-day risk of RBC transfusion was 3.5% among aspirin users versus 1.1% among aspirin nonusers (adjusted RR = 1.9, 95% CI: 1.4–2.7), and 1.8% among SSRI users versus 1.2% among SSRI nonusers (adjusted RR = 1.2, 95% CI: 0.7–1.9). Red blood cell transfusion risk was increased among NSAID users, but not in a sensitivity analysis with a 30-day exposure window. Red blood cell transfusion risk was not increased among SSRI and statin users.

**Conclusions:**

Primary breast cancer surgery confers a low risk of RBC transfusion. Still, use of aspirin and possibly NSAIDs, but not SSRIs and statins, is associated with increased red blood cell transfusion. This increased risk is not explained by differences in age, stage, or comorbidity level.

**Electronic supplementary material:**

The online version of this article (doi:10.1186/s13058-017-0926-2) contains supplementary material, which is available to authorized users.

## Background

Breast cancer is the most common cancer in women and surgery is the primary treatment [1]. Bleeding after primary breast cancer surgery is rare, and occurs in 2% to 4% of patients [2, 3]. However, prescription medicines that affect platelet function may increase the risk of postsurgical bleeding, necessitating transfusion. Nonaspirin nonselective nonsteroidal anti-inflammatory drugs (NSAIDs) and aspirin are frequently prescribed drugs that inhibit cyclooxygenase-1 (Cox-1), thereby preventing platelet aggregation. NSAIDs inhibit coagulation on a short-term basis, while aspirin inhibits clotting for up to 7 days after administration [4, 5]. Use of NSAIDs is a well-established risk factor for gastrointestinal bleeding and also may have an impact on postsurgical bleeding [6–8]. Selective serotonin reuptake inhibitors (SSRIs) decrease platelet serotonin storage and platelet function and are correlated with increased risk of both gastrointestinal and postsurgical bleeding [9–12]. Other frequently used drugs, such as statins, may increase the risk of bleeding through a potential direct antiplatelet effect and an antithrombotic effect unrelated to cholesterol-lowering activity [13, 14].

We have previously studied the association of prescriptions for SSRIs and glucocorticoids and the risk of re-operation due to postsurgical bleeding after breast cancer primary surgery [11, 15]. Previous research has investigated predictors of red blood cell (RBC) transfusion in cardiac and hip fracture surgery patients [16, 17]. However, no previous studies have examined the association of these drugs with the risk of RBC transfusion after breast cancer surgery. Prescription medications represent potentially modifiable risk factors that could be intervened upon to reduce the risk of RBC transfusion. Therefore, in a population-based cohort of Danish breast cancer patients, we investigated the association of prescription use of aspirin, NSAIDs, SSRIs, and statins with the risk of RBC transfusion. Here, we investigated the association of the most frequently used prescription drugs – aspirin, NSAIDs, statins, and SSRIs – with the risk of RBC transfusion as a proxy for postoperative bleeding in a population-based cohort of Danish breast cancer patients.

## Methods

### Study population

We conducted this cohort study in Denmark using population-based registries. Denmark’s National Health Service provides tax-funded health care to all Danish residents, including access to hospital care and partial reimbursement for prescribed medications [18]. The unique civil personal registration (CPR) number assigned to each Danish resident, encodes gender and date of birth, and enables accurate individual-level linkage among population-based registries [19].

The Danish Cancer Registry (DCR) has recorded data on incident cancers since 1943. Voluntary registration became mandatory in 1987. The DCR contains information on the civil registration number, age, gender, cancer diagnosis, method of verification, extent of spread of the tumor at the time of diagnosis, stage, treatment and vital status (date of death and emigration) [20]. The Danish National Patient Registry (DNPR) has maintained records on all nonpsychiatric hospitalizations at the individual patient level since 1977, and on all hospital outpatient and emergency department visits since 1994 [20]. Information is recorded in the DNPR immediately after inpatient discharge or outpatient visit and includes the CPR number, dates of admission and discharge/visit date, and up to 20 diagnoses coded according to the International Classification of Diseases, Tenth Revision (ICD-10) [21]. We used the DCR to identify a cohort of women aged ≥ 35 years with a first-time diagnosis of breast cancer between 2005 and 2012 (see Appendix for diagnostic codes in Additional file 1). We linked the DCR data to the DNPR to retrieve information on primary breast cancer surgery (mastectomy or breast-conserving surgery (BCS)) in the same period (see Appendix for surgery codes in Additional file 1).

As shown in Additional file 2: Figure S1, we identified 31,338 patients with incident breast cancer. Based on diagnoses in the DCR, we excluded 5664 patients with stage IV breast cancer, breast cancer of unknown stage, or a previous cancer diagnosis other than nonmelanoma skin cancer, as these patients may receive RBC transfusions for supportive/palliative care rather than for bleeding. We further excluded patients for whom ≥ 1 month had elapsed between their dates of diagnosis and breast cancer-directed primary surgery in the DCR and DNPR (*n* = 2653). In addition, we excluded patients who redeemed ≥ 1 prescription for antithrombotic drugs (platelet inhibitors, anticoagulants, or novel oral anticoagulants; see Appendix for drug list with Anatomical Therapeutic Classification (ATC) codes in Additional file 1) in the 60 days prior to surgery (*n* = 783), as these patients are likely to have an increased risk of bleeding. The final study population included 22,238 women.

### Prescription data

All pharmacies in Denmark use computerized accounting systems to record patients’ CPR-number and type and quantity of medication dispensed (including tablet quantity, strength, and package sizes). After adding the medication’s ATC system code and defined daily dose, the data is transferred electronically to the National Health Service and Danish National Health Service Prescription Database (DNHSPD) [22]. The DNHSPD has registered all prescriptions redeemed in Denmark since 2004 [23]. We identified prescriptions for the following drug types: aspirin (>99% low-dose aspirin), NSAIDs (only nonselective), SSRIs, and statins (see Appendix for drug list with ATC codes in Additional file 1) [24]. Patients were categorized as nonusers (no prescriptions prior to surgery) and users (if they filled ≥ 1 prescription in the 60 days prior to surgery) of aspirin, NSAIDs, SSRIs, and statins. We chose a 60-day period to define use because these drugs are most often prescribed for 2 months’ duration.

### Data on potential confounders

We used the Charlson Comorbidity Index (CCI) to estimate the extent of comorbid disease diagnosed up to 10 years before breast cancer diagnosis. The CCI scores were categorized into three levels: a score of 0 (low, given to patients with no previous record of diseases included in the CCI); a score of 1–2 (moderate comorbidity); and a score of 3 or more (high comorbidity) [25]. We obtained information on age at breast cancer surgery and primary surgery type from the DNPR according to the Danish Classification of Surgical Procedures and Therapy. All breast cancer patients underwent mastectomy or BCS (see Appendix for surgery codes in Additional file 1). We ascertained data on cancer stage from the DCR and linked DCR data to the DNPR data. Data on age and coexistent diseases at diagnosis were retrieved from the DNPR.

### Outcome

The Danish Transfusion Database (DTDB) was used to ascertain data on the receipt of a RBC transfusion up to 14 days after surgery [26]. The 14-day window ensured that postoperative RBC transfusion was related to the breast cancer surgery rather than to preexisting conditions. Records in the DTDB are generated by linking to other Danish health registers and include patient admission, diagnostics, medication, and medical procedures, with mandatory registration of every transfusion performed in Denmark since 2001. Data on the use of blood components, including the number of blood components transfused, and measurements of hemoglobin in all transfused patients, are considered to be of high quality [20]. Information on preoperative hemoglobin level (measured up to 30 days prior to surgery) and the number and types of blood transfusions was retrieved from the DTDB.

### Statistical methods

We tabulated characteristics of the study population according to aspirin, NSAID, SSRI, and statin use. We calculated the 14-day risk of RBC transfusion among users and nonusers of each drug and used logistic regression models to estimate crude and adjusted odds ratios (ORs) of receipt of postoperative RBC transfusions, with associated 95% confidence intervals (CIs), controlling for confounding by age, cancer stage, and CCI score. We expected RBC transfusion to be a rare event in breast cancer patients, so the ORs provide an estimate of the relative risks (RRs) [27]. We evaluated the interactions between the exposure variables and the potential confounders, and no evidence of effect modification was found. We also calculated the number needed to harm the statistic where relevant (with 95% CIs) [28]. We conducted several sensitivity analyses: restricting postoperative RBC transfusions to those occurring within 7 days after surgery; defining drug use as prescriptions redeemed 1–30 days prior to surgery; adjusting for selected comorbidities (cardiovascular disease, chronic pulmonary disease, and diabetes) instead of using CCI scores; and excluding patients with anemia [hemoglobin concentration < 12 g/dL (7,45 mmol/L)]. We examined the risk of RBC transfusion according to each of the analytic variables, stratified by surgery type, to examine any evidence of effect modification on the multiplicative scale; a two-sided value of *p* < 0.2 was considered statistically significant. All data analyses were performed using SAS version 9.4 (SAS Institute, Inc., Cary, NC, USA).

## Results

Our study included 22,238 women aged ≥ 35 years with incident breast cancer who underwent surgery for their cancer between 2005 and 2012. Table 1 shows baseline characteristics of the cohort according to aspirin, NSAID, SSRI, and statin use. Overall, 45% were aged below 60 years at diagnosis, 85% had a CCI score of 0, and about 70% underwent BCS as primary breast cancer surgery. Over 40% had no preoperative measurement of hemoglobin concentration. There was no notable difference in the characteristics of patients with and without information on preoperative hemoglobin levels. Among women with information available on their preoperative hemoglobin level, about 5% had anemia [< 12 g/dL (7.4 mmol/L)]. Overall 1336 (6.0%) patients used aspirin, 1774 (8.0%) used NSAIDs, 1080 (4.9%) used SSRIs, and 2029 (9.1%) used statins. Compared with nonusers, users of these drugs were older, were more likely to have anemia, and had higher CCI scores. More users of aspirin, NSAIDs, SSRIs, and statins underwent mastectomy compared with nonusers of each drug type (Table 1). The most frequent comorbidities among users and nonusers of the four drugs were chronic pulmonary disease, diabetes, and cerebrovascular disease (see Additional file 2: Table S2).

**Table 1:** Baseline characteristics of 22,238 breast cancer surgery patients according to use of selected prescription drugs, Denmark, 2005–2012

	All patients		Aspirin				NSAIDs				SSRIs				Statins			
			Nonusers		Users		Nonusers		Users		Nonusers		Users		Nonusers		Users	
	N	%	N	%	N	%	N	%	N	%	N	%	N	%	N	%	N	%
Total	22,238	100.0	20,902	100.0	1336	100.0	20,464	100.0	1774	100.0	21,158	100.0	1080	100.0	20,209	100.0	2029	100.0
Age at diagnosis (years)																		
35–49	3794	17.1	3778	18.1	16	1.2	3606	17.6	188	10.6	3648	17.2	146	13.5	3743	18.5	51	2.5
50–59	6102	27.4	5958	28.5	144	10.8	5661	27.7	441	24.9	5806	27.4	296	27.4	5734	28.4	368	18.1
60–69	7831	35.2	7335	35.1	496	37.1	7209	35.2	622	35.1	7467	35.3	364	33.7	6843	33.9	988	48.7
70–79	3233	14.5	2808	13.4	425	31.8	2844	13.9	389	21.9	3051	14.4	182	16.9	2748	13.6	485	23.9
> =80	1278	5.7	1023	4.9	255	19.1	1144	5.6	134	7.6	1186	5.6	92	8.5	1141	5.6	137	6.8
Stage																		
I	10,161	45.7	9580	45.8	581	43.5	9347	45.7	814	45.9	9689	45.8	472	43.7	9192	45.5	969	47.8
II	9587	43.1	8992	43.0	595	44.5	8842	43.2	745	42.0	9118	43.1	469	43.4	8739	43.2	848	41.8
III	2490	11.2	2330	11.1	160	12.0	2275	11.1	215	12.1	2351	11.1	139	12.9	2278	11.3	212	10.4
Charlson comorbidity score																		
0	18,863	84.8	18,131	86.7	732	54.8	17,405	85.1	1458	82.2	18,064	85.4	799	74.0	17,500	86.6	1363	67.2
1–2	3074	13.8	2560	12.2	514	38.5	2782	13.6	292	16.5	2829	13.4	245	22.7	2498	12.4	576	28.4
> =3	301	1.4	211	1.0	90	6.7	277	1.4	24	1.4	265	1.3	36	3.3	211	1.0	90	4.4
Specific comobidities of interest																		
Myocardial infarction	197	0.9	96	0.5	101	7.6	185	0.9	12	0.7	178	0.8	19	1.8	116	0.6	81	4.0
Congestive heart failure	211	0.9	146	0.7	65	4.9	189	0.9	22	1.2	194	0.9	17	1.6	164	0.8	47	2.3
Diabetes I and II	652	2.9	484	2.3	168	12.6	589	2.9	63	3.6	595	2.8	57	5.3	392	1.9	260	12.8
Diabetes with end organ damage	239	1.1	159	0.8	80	6.0	214	1.0	25	1.4	212	1.0	27	2.5	142	0.7	97	4.8
Pre-operative hemoglobin concentration																		
No data	9137	41.1	8603	41.2	534	40.0	8401	41.1	736	41.5	8672	41.0	465	43.1	8308	41.1	829	40.9
< 7.4 mmol/L	660	3.0	578	2.8	82	6.1	577	2.8	83	4.7	602	2.8	58	5.4	578	2.9	82	4.0
> =7.4 mmol/L	12,441	55.9	11,721	56.1	720	53.9	11,486	56.1	955	53.8	11,884	56.2	557	51.6	11,323	56.0	1118	55.1
Type of primary surgery																		
Mastectomy	6935	31.2	6406	30.6	529	39.6	6324	30.9	611	34.4	6551	31.0	384	35.6	6300	31.2	635	31.3
Breast-conserving surgery	15,303	68.8	14,496	69.4	807	60.4	14,140	69.1	1163	65.6	14,607	69.0	696	64.4	13,909	68.8	1394	68.7

*NSAID*s nonsteroidal anti-inflammatory drugs, *SSRIs* selective serotonin reuptake inhibitors

Overall, 279 (1.3%) women received at least one RBC transfusion within 14 days of surgery. Risk of receiving a RBC transfusion, according to prescriptions redeemed for aspirin, NSAIDs, SSRIs, and statins, are presented in Table 2. The risk of RBC transfusion was 3.5% among aspirin users versus 1.1% among aspirin nonusers (corresponding to an adjusted OR of 1.9, 95% CI: 1.4–2.7). The increased risk of RBC transfusion among aspirin users was unchanged in sensitivity analyses (see Additional file 2: Tables S2–S5). The number needed to harm (NNH) based on ≥ 1 prescription redeemed in the 60 days prior to surgery was 41.5 (95% CI: 29.4–70.9) for aspirin. Although we observed a slightly elevated risk of RBC transfusion among NSAID users compared with NSAID nonusers (adjusted OR = 1.4, 95% CI: 0.9–2.0), this association attenuated in sensitivity analysis (see Additional file 2: Table S3). The risks of RBC transfusion did not differ for users of SSRIs (adjusted OR = 1.2, 95% CI: 0.7–1.9) or statins (adjusted OR = 1.0, 95% CI: 0.7–1.4) compared with nonusers.

**Table 2:** Risk and crude and adjusted odds ratios for blood transfusion within 14 days of surgery among 22,238 breast cancer patients, according to use of selected prescription drugs

	Transfused patients, N	All patients, N	Risk (%)	Crude OR (95% CI)	Adjusted OR^*^ (95% CI)
				Estimate	Estimate
Aspirin					
Nonusers	232	20,902	1.1	1.0 (ref)	1.0
Users	47	1336	3.5	3.2 (2.4–4.5)	1.9 (1.4–2.7)
NSAIDs					
Nonusers	247	20,464	1.2	1.0	1.0
Users	32	1774	1.8	1.5 (1.0–2.2)	1.4 (0.9–2.0)
SSRIs					
Nonusers	260	21,158	1.2	1.0	1.0
Users	19	1080	1.8	1.4 (0.9–2.3)	1.2 (0.7–1.9)
Statins					
Nonusers	248	20,209	1.2	1.0	1.0
Users	31	2029	1.5	1.2 (0.9–1.8)	1.0 (0.7–1.4)

*NSAID*s nonsteroidal anti-inflammatory drugs, *SSRIs* selective serotonin reuptake inhibitors

*Odds ratio (OR) adjusted for age, cancer stage, and Charlson Comorbidity Index score

Table 3 shows the risk of RBC transfusion according to prescriptions for each of the exposure drugs, stratified by surgery type. We found no evidence of multiplicative interaction between the estimates for mastectomy and breast-conserving surgery.

**Table 3:** Risk and crude and adjusted odds ratios for blood transfusion within 14 days of surgery among 22,238 breast cancer patients, according to use of selected prescription drugs and stratified by surgery type

	Mastectomy					Breast-conserving surgery					
	Transfused patients, N	All patients, N	Risk (%)	Crude OR (95% CI)	Adjusted OR* (95% CI)	Transfused patients, N	All patients, N	Risk (%)	Crude OR (95% CI)	Adjusted OR* (95% CI)	
Aspirin											
Non-users	178	6406	2.8	1.0 (ref)	1.0	54	14,496	0.4	1.0	1.0	*p* = 0.87
Users	37	529	7.0	2.6 (1.8–3.8)	1.9 (1.3–2.9)	10	807	1.2	3.4 (1.7– 6.6)	1.8 (0.9–3.8)	
NSAIDs											
Non-users	187	6324	3.0	1.0	1.0	60	14,140	0.4	1.0	1.0	*p* = 0.23
Users	28	611	4.6	1.6 (1.1–2.4)	1.4 (1.0–2.2)	4	1163	0.3	0.8 (0.3–2.2)	0.7 (0.3–2.0)	
SSRIs											
Non-users	198	6551	3.0	1.0	1.0	62	14,607	0.4	1.0	1.0	*p* = 0.23
Users	17	384	4.4	1.5 (0.9–2.5)	1.3 (0.8–2.2)	2	696	0.3	0.7 (0.2– 2.8)	0.5 (0.1–2.2)	
Statins											
Non-users	189	6300	3.0	1.0	1.0	59	13,909	0.4	1.0	1.0	*p* = 0.29
Users	26	635	4.1	1.4 (0.9–2.1)	1.1 (0.7–1.7)	5	1394	0.4	0.8 (0.3–2.1)	0.6 (0.2–1.6)	

*NSAID*s nonsteroidal anti-inflammatory drugs, *SSRIs* selective serotonin reuptake inhibitors

*Odds ratio (OR) adjusted for age, cancer stage and Charlson Comorbidity Index score

## Discussion

Primary breast cancer surgery among women with nonmetastatic breast cancer was associated with low risk of RBC transfusion. Our findings suggest that use of aspirin correlates with an increased risk of RBC transfusion. For every 42 women who filled ≥ 1 prescription in the 60 days prior to surgery, one patient underwent RBC transfusion within 14 days after surgery. We also found a potentially higher risk of RBC transfusion among NSAID users, compared with nonusers of these drugs. The risk of RBC transfusion did not differ among SSRI and statin users compared with nonusers.

Several factors should be considered when interpreting our results. Study strengths include its population-based registry setting, with availability of complete prescription and follow-up data. This reduced the risk of misclassification due to differential loss to follow-up. The unique CPR number facilitated accurate individual-level linkage across the Danish registry network. Use of prospectively collected data from prescription records, whose completeness approaches 100%, ensured an unbiased assessment of exposure before breast cancer diagnosis and eliminated recall bias [29]. We had access to comprehensive information on potential confounders, including comorbid diseases.

We had no information on prescription compliance. Still, noncompliance would result in misclassification of nonusers as users and therefore could not account for any increased risk of RBC transfusion related to drug use. Some women defined as users may not have taken their medication right up to the day of surgery, however, misclassification would bias our findings to the null. Another concern is that our information on prescription drug exposure relied entirely on registration of dispensed prescriptions rather than actual consumption of the pills. However, as patients have to pay a portion of the cost of their redeemed prescriptions, our estimates are likely to reflect actual drug use. Over-the-counter use of aspirin and NSAIDs is an additional concern, because this is not covered by registry data. However, over-the-counter drug use would result in misclassification of users as nonusers and again bias our findings to the null. Research has suggested that nonprescription use of low-dose aspirin accounts for only 8% of all low-dose aspirin use in Denmark [30]. As patients are reimbursed for a portion of the cost of prescribed medicines, long-term and continuous use of aspirin is likely to be via prescription. We had no information on medication use during hospitalization. However, breast cancer surgery is primarily an elective procedure, with a short amount of time from hospital admission to surgery (0.57 days in our study population). Thus patients are likely to be vulnerable to the effects of medication taken prior to hospitalization.

We used a “prevalent user design”, comparing current users with nonusers of the exposure drugs. This design may have introduced selection bias via a “healthy user effect”. Individuals who adhere to preventive medication may be more likely to see their doctor on a regular basis, stop smoking, exercise, eat a healthy diet, and have more engagement with the health-care system resulting in better outcomes. This has been seen in studies of statin use with large protective effects [31]. To address this potential healthy user bias, we restricted the study population to new users of the prescription drugs (those with a first prescription for the exposure drugs up to 60 days prior to breast cancer primary surgery). However, the number of patients transfused in each group was too low to provide meaningful estimates. Nonetheless, we note that previous research suggests that Danish statin users are not healthier than the background population [32].

While we adjusted for comorbidities, we had no information on the severity of conditions included in the CCI. We also lacked information on comorbid conditions diagnosed in primary care, but which may not be sufficiently severe to warrant a hospital diagnosis. These limitations may have resulted in residual confounding. Our findings may also be prone to confounding by indication. Unknown or unmeasured factors related to clinical characteristics or medical conditions may have triggered aspirin use, and at the same time, increased the risk of RBC transfusion. We note that aspirin users had a higher frequency of specific comorbidities including myocardial infarction, congestive heart failure, and diabetes. Cardiovascular disease (CVD) is a major indication for low-dose aspirin use, and the threshold for RBC transfusion is lower in patients with cardiovascular disease [33]. However, CVD also correlates with statin use, which was not associated with an increased risk of RBC transfusion in our study, so confounding by indication is likely minimal. We had no registry data on lifestyle factors. Therefore, unmeasured confounding could contribute to the association between use of aspirin, NSAIDs, SSRIs, or statins and the risk of postoperative RBC transfusion if lifestyle factors differed between breast cancer patients who were users versus nonusers.

We had no data on the extent of postoperative bleeding and preoperative hemoglobin levels in over 40% of patients due to missing data in the DTDB. However, we note similar characteristics of patients with and without information on preoperative hemoglobin levels (data not presented). We could have imputed missing hemoglobin data, but hemoglobin may be an intermediate factor in the association between use of prescription drugs and the risk of postoperative RBC transfusion. We excluded 2653 patients who did not undergo surgery within 1 month of diagnosis. Thus patients who underwent neo-adjuvant chemotherapy were also excluded. Therefore, our findings may not be generalizable to all nonmetastatic breast cancer patients.

To the best of our knowledge, this is the first population-based study to investigate the use of common prescription drugs and the risk of postoperative RBC transfusion after primary breast cancer surgery. Studies have shown that aspirin use increases the risk of postoperative bleeding and possibly the need for blood transfusion, and that it depends on extent of surgery in addition to patient factors [34, 35]. Studies across multiple surgical specialties, including heart surgery, surgery for gastric cancer, and colonoscopic polypectomy, also have reported increased risk of blood transfusion associated with aspirin use [34–36]. However, other studies suggest no increased risk of bleeding associated with preoperative aspirin use among patients undergoing nephrectomy, prostate cancer surgery, and lung cancer surgery [37–39]. Our findings also contrast with those from the STRATAGEM clinical trial, which randomized 291 patients to aspirin or placebo for noncardiac elective surgeries. STRATAGEM found no evidence of increased bleeding risk associated with aspirin use, but breast cancer surgery was not included in the trial [40].

Although our overall analyses suggested an elevated risk of postoperative RBC transfusion among users of NSAIDs, this was not evident in sensitivity analyses. This finding concurs with two previous meta-analyses – one investigating the association of NSAIDs with the risk of postoperative bleeding in patients undergoing plastic surgery [7], and the other, focused on the association of NSAIDs with bleeding risk after tonsillectomy [8].

We observed little evidence of an association between SSRI use and postoperative RBC transfusion. This agrees with previous studies on patients undergoing coronary artery bypass surgery (CABG), which reported that use of preoperative SSRIs was not associated with any substantial risk of blood transfusion and bleeding [41, 42]. Nonetheless, this finding seems at odds with our previous research, showing an increased risk of reoperation due to postsurgical bleeding in breast cancer patients [11]. It also contrasts with the increased risk of bleeding events associated with SSRI use among patients undergoing breast biopsy [43]. Taken together, this suggests that SSRIs may correlate with bleeding, but are unlikely to induce bleeding severe enough to warrant a blood transfusion.

We did not find an association between statin use and postoperative RBC transfusion. To our knowledge, no previous studies have investigated this association. Our findings agree with those observed among patients with gastrointestinal hemorrhage or bleeding in general, although we note that statin therapy has been associated with increased risk of spontaneous intracerebral hemorrhage [44–46].

Breast cancer surgery is soft tissue surgery, and often characterized by extensive dissection, increasing the risk of blood loss. Although RBC transfusion is rare, it induces substantial morbidity and can delay further cancer-directed treatment [11, 47]. The balance between the risk of bleeding associated with aspirin, and thrombotic events due to aspirin withdrawal, should be assessed carefully when choosing a surgical procedure appropriate for an individual patient. Our findings, therefore, may help to guide decision making and counseling for breast cancer patients who use prescription medications.

## Conclusions

Primary breast cancer surgery confers a low risk of RBC transfusion. Still, use of aspirin, and possibly NSAIDs, but not SSRIs and statins, is associated with increased transfusion risk. The increased risk is not explained by differences in age, cancer stage, or comorbidity level.

## Additional files

Additional file 1: Appendix. Diagnostic codes, drug list with ATC codes and surgery codes. (DOCX 16 kb)

Additional file 2: Supplementary Figure S1 and Supplementary Tables S1–S5. **Figure S1.** Flow diagram. **Table S1.** Specific comorbid conditions included in the Charlson Comorbidity Index, according to use of selected prescription drugs. **Table S2.** Risk and crude and adjusted odds ratios for blood transfusion within 7 days of surgery among 22,238 breast cancer patients, according to use of selected prescription drugs. **Table S3.** Risk and crude and adjusted odds ratios for postoperative blood transfusion within 14 days of surgery among 22,238 breast cancer patients, according to use of selected prescription drugs and with the exposure window defined as 1–30 days before surgery. **Table S4.** Risk and crude and adjusted odds ratios for postoperative blood transfusion within 14 days of surgery among 22,238 breast cancer patients, according to use of selected prescription drugs and adjusted for selected comorbidities (cardiac disease, chronic pulmonary disease, and diabetes). **Table S5.** Risk and crude and adjusted odds ratios for postoperative blood transfusion within 14 days of surgery among 21,578 breast cancer patients according to use of selected prescription drugs, with the exposure window defined as 1–30 days before surgery and after excluding patients with anemia [< 12 g/dL (7.4 mmol/L)]. (ZIP 346 kb)

## Abbreviations

ATC: Anatomical Therapeutic Classification
BCS: Breast-conserving surgery
CABG: Coronary artery bypass surgery
CCI: Charlson Comorbidity Index
CIs: Confidence intervals
Cox-1: Cyclooxygenase-1
CPR: Civil personal registration
CVD: Cardiovascular disease
DCR: The Danish Cancer Registry
DNPR: The Danish National Patient Registry
DTDB: The Danish Transfusion Database
NNH: Number needed to harm
NSAIDs: Nonsteroidal anti-inflammatory drugs
OR: Odds ratio
RBC: Red blood cell
RR: Relative risk
SSRIs: Selective serotonin reuptake inhibitors
