# Supplementary material for: Use of prescription drugs and risk of postoperative red blood cell transfusion in breast cancer patients: a Danish population-based cohort study
Source: Breast Cancer Res. 2017 Dec 22;19:135. doi: 10.1186/s13058-017-0926-2 (PMC5741918; doi:10.1186/s13058-017-0926-2)
Supplement: Supplementary file 1 — Diagnostic codes, drug list with ATC codes and surgery codes. (DOCX 16 kb) [file 13058_2017_926_MOESM1_ESM.docx]

**Appendix**

***Diagnostic codes***

Breast cancer (ICD-10 codes: C50.0-50.6, C50.8, and C50.9)

***Drug list with ATC codes***

NSAIDs

Aspirin: [Acetylsalicylsyre](https://pro.medicin.dk/Medicin/Indholdsstoffer/77) (B01AC06), acetylsalicylsyre (N02BA01), and acetylsalicylsyre, caffein (N02BA51)

NSAIDs: Dexibuprofen (M01AE14), etodolac (M01AB08), ibuprofen (M01AE01), ketorolac (M01AB15), lornoxicam (M01AC05), nabumeton (M01AX01), naproxen (M01AE02), phenylbutazon (M01AA01), piroxicam (M02AA07), tenoxicam (M01AC02), tiaprofensyre (M01AE11) and tolfenamsyre (M01AG02)

SSRIs

Fluoxetine (N06AB03), citalopram (N06AB04), paroxetine (N06AB05), sertraline (N06AB06), fluvoxamine (N06AB08), and escitalopram (N06AB10)

Statins

Lipophilic statins: simvastatin (C10AA01), lovastatin (C10AA02), fluvastatin (C10AA04), and cerivastatin (C10AA06). Hydrophilic statins: atorvastatin (C10AA05), pravastatin (C10AA03), and rosuvastatin (C10AA07)

Antithrombotic drugs

Warfarin (B01AA03), phenprocoumon  (B01AA04), antitrombin (B01AB02), delteparin (B01AB04), enoxaparin (B01AB05), reviparin (B01AB08), tinzaparin (B01AB10), clopidogrel (B01AC04), dipyridamol (B01AC07), epoprostenol (B01AC09), abciximab (B01AC13), anagrelide (B01AC14), [eptifibatid](http://pro.medicin.dk/Medicin/Indholdsstoffer/3057) (B01AC16), tirofiban (B01AC17), acetylsalicylic acid and dipyridamol (B01AC30), streptokinase (B01AD01), alteplase (B01AD02), urokinase (B01AD04), reteplase (B01AD07), drotrecogin alfa (B01AD10), [tenecteplase](http://pro.medicin.dk/Medicin/Indholdsstoffer/3118) (B01AD11), melagatran (B01AE04), ximelagatran (B01AE05), [dabigatranetexilat](http://pro.medicin.dk/Medicin/Indholdsstoffer/3366) (B01AE07), [rivaroxaban](http://pro.medicin.dk/Medicin/Indholdsstoffer/3381) (B01AF01), [apixaban](http://pro.medicin.dk/Medicin/Indholdsstoffer/3517) (B01AF02), [edoxaban](http://pro.medicin.dk/Medicin/Indholdsstoffer/3725) (B01AF03), lepirudin (B01AX03), and [fondaparinux](http://pro.medicin.dk/Medicin/Indholdsstoffer/3157) (B01AX05)

***Surgery codes***

Mastectomy (code KHAC)

Breast-conserving surgery (code KHAB)
