# Supplementary material for: Use of prescription drugs and risk of postoperative red blood cell transfusion in breast cancer patients: a Danish population-based cohort study
Source: Breast Cancer Res. 2017 Dec 22;19:135. doi: 10.1186/s13058-017-0926-2 (PMC5741918; doi:10.1186/s13058-017-0926-2)
Supplement: Supplementary file 2 — Supplementary Figure S1 and Supplementary Tables S1–S5. Figure S1. Flow diagram. Table S1. Specific comorbid conditions included in the Charlson Comorbidity Index, according to use of selected prescription drugs. Table S2. Risk and crude and adjusted odds ratios for blood transfusion within 7 days of surgery among 22,238 breast cancer patients, according to use of selected prescription drugs. Table S3. Risk and crude and adjusted odds ratios for postoperative blood transfusion within 14 days of surgery among 22,238 breast cancer patients, according to use of selected prescription drugs and with the exposure window defined as 1–30 days before surgery. Table S4. Risk and crude and adjusted odds ratios for postoperative blood transfusion within 14 days of surgery among 22,238 breast cancer patients, according to use of selected prescription drugs and adjusted for selected comorbidities (cardiac disease, chronic pulmonary disease, and diabetes). Table S5. Risk and crude and adjusted odds ratios for postoperative blood transfusion within 14 days of surgery among 21,578 breast cancer patients according to use of selected prescription drugs, with the exposure window defined as 1–30 days before surgery and after excluding patients with anemia [< 12 g/dL (7.4 mmol/L)]. (ZIP 346 kb) [file 13058_2017_926_MOESM2_ESM.zip › Table S4 in Additional file 2.docx]

**Table S4** Risk and crude and adjusted odds ratios for postoperative blood transfusion within 14 days of surgery among 22,238 breast cancer patients, according to use of selected prescription drugs and adjusted for selected comorbidities (cardiac disease, chronic pulmonary disease, and diabetes).

|  |  |  |  | **Crude OR (95% CI)** | **Adjusted OR^*^ (95% CI)** |
| --- | --- | --- | --- | --- | --- |
|  | Transfused  patients, N | All patients,  N | Risk (%) | Estimate | Estimate |
| **Aspirin**  Non-users  Users | 232  47 | 20902  1336 | 1.1  3.5 | 1.0 (ref)  3.2 (2.4, 4.5) | 1.0  1.9 (1.3, 2.6) |
| **NSAIDs**  Non-users  Users | 247  32 | 20464  1774 | 1.2  1.8 | 1.0  1.5 (1.0, 2.2) | 1.0  1.4 (1.0, 2.0) |
| **SSRIs**  Non-users  Users | 260  19 | 21158  1080 | 1.2  1.8 | 1.0  1.4 (0.9, 2.3) | 1.0  1.2 (0.7, 1.9) |
| **Statins**  Non-users  Users | 248  31 | 20209  2029 | 1.2  1.5 | 1.0  1.2 (0.9, 1.8) | 1.0  1.0 (0.6, 1.4) |

*Odds ratio (OR) adjusted for age, cancer stage and Charlson Comorbidity Index score.
