# Supplementary material for: Use of prescription drugs and risk of postoperative red blood cell transfusion in breast cancer patients: a Danish population-based cohort study
Source: Breast Cancer Res. 2017 Dec 22;19:135. doi: 10.1186/s13058-017-0926-2 (PMC5741918; doi:10.1186/s13058-017-0926-2)
Supplement: Supplementary file 2 — Supplementary Figure S1 and Supplementary Tables S1–S5. Figure S1. Flow diagram. Table S1. Specific comorbid conditions included in the Charlson Comorbidity Index, according to use of selected prescription drugs. Table S2. Risk and crude and adjusted odds ratios for blood transfusion within 7 days of surgery among 22,238 breast cancer patients, according to use of selected prescription drugs. Table S3. Risk and crude and adjusted odds ratios for postoperative blood transfusion within 14 days of surgery among 22,238 breast cancer patients, according to use of selected prescription drugs and with the exposure window defined as 1–30 days before surgery. Table S4. Risk and crude and adjusted odds ratios for postoperative blood transfusion within 14 days of surgery among 22,238 breast cancer patients, according to use of selected prescription drugs and adjusted for selected comorbidities (cardiac disease, chronic pulmonary disease, and diabetes). Table S5. Risk and crude and adjusted odds ratios for postoperative blood transfusion within 14 days of surgery among 21,578 breast cancer patients according to use of selected prescription drugs, with the exposure window defined as 1–30 days before surgery and after excluding patients with anemia [< 12 g/dL (7.4 mmol/L)]. (ZIP 346 kb) [file 13058_2017_926_MOESM2_ESM.zip › Table S5 in Additional file 2.docx]

**Table S5** Risk and crude and adjusted odds ratios for postoperative blood transfusion within 14 days of surgery among 21,578 breast cancer patients, according to use of selected prescription drugs, with the exposure window defined as 1-30 days before surgery and after excluding patients with anemia (<12g/dL (7.4 mmol/l)).

|  |  |  |  | **Crude OR (95% CI)** | **Adjusted OR^*^ (95% CI)** |
| --- | --- | --- | --- | --- | --- |
|  | Transfused  patients, N | All patients,  N | Risk (%) | Estimate | Estimate |
| **Aspirin**  Non-users  Users | 137  29 | 20324  1254 | 0.7  2.3 | 1.0 (ref)  3.5 (2.3, 5.2) | 1.0  1.9 (1.2, 2.9) |
| **NSAIDs**  Non-users  Users | 151  15 | 19887  1691 | 0.8  0.9 | 1.0  1.2 (0.7, 2.0) | 1.0  1.0 (0.6, 1.8) |
| **SSRIs**  Non-users  Users | 158  8 | 20556  1022 | 0.8  0.8 | 1.0  1.0 (0.5, 2.1) | 1.0  0.8 (0.4, 1.7) |
| **Statins**  Non-users  Users | 147  19 | 19631  1947 | 0.7  1.0 | 1.0  1.3 (0.8, 2.1) | 1.0  1.0 (0.6, 1.6) |

*Odds ratio (OR) adjusted for age, cancer stage and Charlson Comorbidity Index score.
